# Prenatal diagnosis and molecular characterization of *PKHD1* variants in two Chinese fetuses with Caroli disease/syndrome

**DOI:** 10.3389/fgene.2025.1651306

**Published:** 2025-09-30

**Authors:** Hui Huang, Sheng Zhao, Dan Wang, Shengbao Pan, Fang Liu, Peiwen Chen, Xinlin Chen

**Affiliations:** ^1^ Department of Ultrasonography, Maternal and Child Health Hospital of Hubei Province, Wuhan, Hubei, China; ^2^ Department of Radiology, Maternal and Child Health Hospital of Hubei Province, Wuhan, Hubei, China

**Keywords:** Caroli disease, Caroli syndrome, autosomal recessive polycystic kidney disease, *PKHD1* gene, fetus, prenatal diagnosis, whole exome sequencing, minigene assay

## Abstract

**Background:**

Caroli disease (CD) and Caroli syndrome (CS) are rare inherited disorders characterized by dilatation of intrahepatic bile ducts, caused by *PKHD1* pathogenic variants. Prenatal diagnosis of CD or CS is extremely rare, with only two cases having genetic analysis worldwide. In this study, we describe the prenatal imaging and genetic findings in two Chinese fetuses with CD/CS.

**Methods:**

Prenatal ultrasound and magnetic resonance imaging (MRI) findings were collected for both fetuses. Whole exome sequencing was performed in family 1 and fetus 2, using fetal umbilical cord and parental peripheral blood. The candidate variants were validated using Sanger sequencing. The effect of the splice site variant was evaluated by *in vitro* minigene assays with the pcMINI-C and pcMINI vectors.

**Results:**

Both fetuses presented with multiple dilated intrahepatic bile ducts and features consistent with autosomal recessive polycystic kidney disease (ARPKD) on ultrasound and MRI at 33 weeks (Fetus 1) and 39 weeks (Fetus 2) gestation; Fetus 2 also exhibited oligohydramnios. Trio WES analysis revealed two compound heterozygous variants of *PKHD1*, a missense variant c.7912T>A (p.Tyr2638Asn) and an intronic splice-site variant c.3364 + 3A>T, in Fetus 1, with the father carrying c.7912T>A and the mother c.3364 + 3A>T. WES analysis in Fetus 2 identified two *PKHD1* candidate variants, c.9901G>T (p.Glu3301Ter) and c.2507T>C (p.Val836Ala). These variants were confirmed by Sanger sequencing, and *in silico* prediction and conservation analysis suggested their potential pathogenicity. The c.3364 + 3A>T variant has been previously reported postnatally but functionally uncharacterized. *In vitro* minigene assays demonstrated that it caused exon 29 skipping, leading to a frameshift and a premature stop codon (c.3229_3364del p.Gly1077Alafs*12).

**Conclusion:**

We reported the first prenatally diagnosed CD/CS cases with genetic analysis in the Chinese population, and experimentally validated the pathogenicity of the recurrent splice site variant c.3364 + 3A>T by a minigene assay. Our findings broaden the *PKHD1* variation spectrum in these rare cases and recommend including prenatal cases to refine the reported genotype-phenotype correlations. We also emphasize the need of WES in probands with CD or CS for early molecular diagnosis in subsequent pregnancies.

## 1 Introduction

Caroli disease is a rare inherited disorder characterized by nonobstructive saccular or fusiform dilatation of the intrahepatic bile ducts (Caroli et al., 1958; Castro et al., 2017). It results from the complete or partial arrest of ductal plate remodeling during embryonic development, leading to malformation of the intrahepatic biliary tree (Desmet, 1998; Sgro et al., 2004). The condition is primarily inherited in an autosomal recessive pattern, although autosomal dominant inheritance has also been reported (Mousson et al., 1997; Habib et al., 2006; Hao et al., 2014). Caroli disease is typically diagnosed before the age of 30 with symptoms such as recurrent cholangitis, jaundice, biliary cirrhosis, portal hypertension, and, in some cases, bile duct carcinoma (Habib et al., 2006). Additionally, it is frequently associated with renal abnormalities, including autosomal dominant and recessive polycystic kidney disease, medullary sponge kidney, and medullary cystic disease (Yonem and Bayraktar, 2007).

Two distinct forms of this disease have been identified: type I (Caroli disease, CD), characterized solely by saccular intrahepatic bile duct dilatation, and type II (Caroli syndrome, CS), which is also associated with congenital hepatic fibrosis (CHF) (Desmet, 1998; Kyalwazi et al., 2025). The prevalence of Caroli disease is estimated to be around 1 case per 1,000,000 individuals (Veigel et al., 2009), while Caroli syndrome occurs more frequently, with an estimated incidence of 1 in 10,000 live births (Castro et al., 2020). Due to their overlapping clinical and pathological features, CD and CS are often considered as different developmental stages of the same disease (Taylor and Palmer, 1998; Sgro et al., 2004).

The genetic basis of CD or CS involves mutations in the polycystic kidney and hepatic disease gene 1 (*PKHD1*) (Courcet et al., 2015; Kyalwazi et al., 2025). Located on chromosome 6p12, the *PKHD1* gene comprises at least 86 exons, with the longest open-reading frame transcript consisting of 67 exons. This longest transcript encodes the Fibrocystin/Polyductin (FPC), a 4074-amino-acid protein crucial for collecting duct and biliary differentiation (Onuchic et al., 2002). Mutations in *PKHD1* are also responsible for autosomal recessive polycystic kidney disease (ARPKD), which often co-occurs with CD or CS. To date, over 700 *PKHD1* mutations have been reported (Giacobbe et al., 2022), most of which are associated with ARPKD, while only a small subset has been linked to CD or CS (Courcet et al., 2015; Giacobbe et al., 2022).

Prenatal diagnosis of CD or CS is exceedingly rare. Since the first reported case by Hussman et al. (1991), only six cases have been documented (Hussman et al., 1991; Yuksel et al., 2002; Sgro et al., 2004; Castro et al., 2017; Rivas et al., 2019; Castro et al., 2020). Most of these fetuses either died *in utero* or within days to months after birth. Genetic analysis was performed in only two of these cases, both of which revealed *PKHD1* mutations. Sgro et al. reported a fetus with Caroli disease carrying two compound heterozygous variants, IVS55 + 1G → A and W2690R, in *PKHD1* (Sgro et al., 2004). Rivas et al. described another fetus with Caroli syndrome and ARPKD harboring a single *PKHD1* variant, c.8407T>C (p.Cys2803Arg) (Rivas et al., 2019).

In this study, we present the imaging characteristics of two Chinese cases with prenatally diagnosed CD/CS, using ultrasound and magnetic resonance imaging (MRI). Additionally, we performed whole-exome sequencing (WES) and minigene assays to investigate the genetic basis of these cases. To our knowledge, this is the first report of prenatal diagnosis and genetic analysis of CD or CS in Chinese population. Our findings expand the spectrum of *PKHD1* variants associated with CD or CS and provide valuable insights for genetic counseling and prenatal diagnosis.

## 2 Materials and methods

### 2.1 Patients

Two fetuses with intrahepatic bile duct dilation detected by ultrasonography or MRI were recruited from the Maternal and Child Health Hospital of Hubei Province between 2018 and 2022. The parents were non-consanguineous, in good health, and had no family history of liver or kidney anomalies. The pregnant women reported no history of adverse drug or environmental exposure during pregnancy. This study was approved by the Ethics Committee of the Maternal and Child Health Hospital of Hubei Province ([2018] IEC BL003), and written informed consent was obtained from the parents.

### 2.2 Whole-exome sequencing (WES) analysis

Umbilical cord samples (2 cm from the fetal side) from the fetuses and peripheral venous blood samples (2 mL each, anticoagulated with EDTA) from the parents of Family 1 were collected. Genomic DNA was extracted using the Magnetic Universal Genomic DNA Kit (TIANGEN, China). DNA quality and quantity were assessed by 1% agarose gel electrophoresis and a Qubit 3.0 fluorometer (Life Technologies, Paisley, UK). WES was performed at BGI (Shenzhen, China), with Trio-WES for Family 1 and Proband-WES for Family 2. Briefly, genomic DNA was sheared by sonication, and fragments of 200–300 bp were selected. After end repair and A-tailing, DNA libraries were prepared using the MGIEasy Universal DNA Library Prep Kit (MGITech). Exome capture was performed using the Roche KAPA HyperExome kit according to the manufacturer’s instructions, followed by sequencing on the MGISEQ-2000 platform (BGI, Shenzhen, China). The sequencing metrics for all samples were as follows: average depth in the target area ≥180X, and >95% of target loci covered at >20X. Raw reads were processed to remove low-quality and adapter-contaminated sequences. Subsequently, clean reads were aligned to the human reference genome GRCh37/hg19 using the Burrows–Wheeler Aligner (BWA; v.0.7.12). Single nucleotide variants (SNVs) and insertions/deletions (indels) were called using the Genome Analysis Toolkit (GATK; v.3.8) and annotated using ANNOVAR software. Variants were filtered based on public population frequency databases (1000 Genomes Project, ESP6500, ExAC, gnomAD) and disease databases (OMIM, HGMD, ClinVar). Synonymous variants and those with a minor allele frequency (MAF) > 0.01 were discarded. The biological effects of variants were predicted using *in silico* tools, including SIFT, PolyPhen2, MutationTaster, Provean, and REVEL. Additionally, splice site variants were evaluated using dbscSNV and SpliceAI. Variant pathogenicity was classified according to the American College of Medical Genetics and Genomics (ACMG) and the Association for Molecular Pathology (AMP) guidelines (Richards et al., 2015). Clinical information and scientific literature were also considered during variant interpretation.

### 2.3 Sanger sequencing and conservation analysis

Candidate causative variants identified by WES were validated using Sanger sequencing in Family 1 and Fetus 2. Primers were designed using Primer Premier 5.0 (Supplementary Table S1). Conservation analysis of the variants across species was performed using MEGA11 (Tamura et al., 2021). Reference protein sequences were obtained from UniProt (Consortium, 2024), including P08F94 (*Homo sapiens*), H2QT63 (*Pan troglodytes*), E2RK30 (*Canis lupus* familiaris), E9PZ36 (*Mus musculus*), A0A8V0XUX1 (Gallus gallus), and A0A6I8QI87 (*Xenopus* tropicalis).

### 2.4 Minigene assay

The biological impact of the candidate splice site variant in the *PKHD1* gene (c.3364 + 3A>T) was assessed using additional *in silico* tools, including varSEAK and MaxEntScan. To validate the in silico-predicted effect of the *PKHD1* variant c.3364 + 3A>T on splicing, *in vitro* minigene assays were performed using the pcMINI-C and pcMINI vectors, as previously described (Fan et al., 2022; Yao et al., 2022). Briefly, a wild-type genomic fragment of *PKHD1*, including partial intron28 (518 bp)-Exon29 (136 bp)-partial intron29 (757 bp)-Exon30 (196 bp), was amplified from the father’s genomic DNA. The variant c.7912T>A was introduced using site-directed mutagenesis. The wild-type (WT) and mutant-type (MT) PCR products were cloned into the pcMINI-C vector at the KpnI and BamHI restriction sites, generating the minigene constructs pcMINI-C-*PKHD1*-WT/MT. Similarly, the minigene constructs pcMINI-*PKHD1*-WT/MT were generated by inserting a shorter fragment, partial intron 28 (278 bp)-Exon 29 (136 bp)- partial intron 29 (466 bp), into the pcMINI vector. Both WT and MT plasmids were confirmed by Sanger sequencing (Figure 5A; Supplementary Figure S1A) and transfected into HEK293T and HepG2 cells using Lipofectamine^®^ 2000 (Thermo Fisher Scientific, Waltham, MA, USA). After 48 h, total RNA was extracted using the Trizol RNAiso PLUS kit (TaKaRa, 9109). Reverse transcription was performed using Hifair^®^ Ⅱ first Strand cDNA Synthesis SuperMix for qPCR (gDNA digester plus) (11123ES60, YEASEN, Shanghai, China), and the resulting cDNA was amplified by PCR. PCR products were analyzed by 1.5% agarose gel electrophoresis and Sanger sequencing. Primers used in this assay are listed in Supplementary Tables S2, S3.

### 2.5 Literature review

To retrieve the reported cases with prenatally diagnosed CD or CS, the literature search was performed in the PubMed, Web of Science, and Google Scholar to identify full-text articles published in English up to March 2025. The key search items included prenatal, fetus, Caroli, and *PKHD1*, combined with Boolean operators as appropriate. Additionally, the reference lists of retrieved articles were manually screened to identify other potentially relevant publications. The clinical and genetic characteristics of the patients in the included studies were collected. Bioinformatic functional predictions and evolutionary conservation analysis of the reported *PKHD1* variants were also performed as mentioned above.

## 3 Results

### 3.1 Patient and sonographic findings

#### 3.1.1 Family 1

**FIGURE 1:**
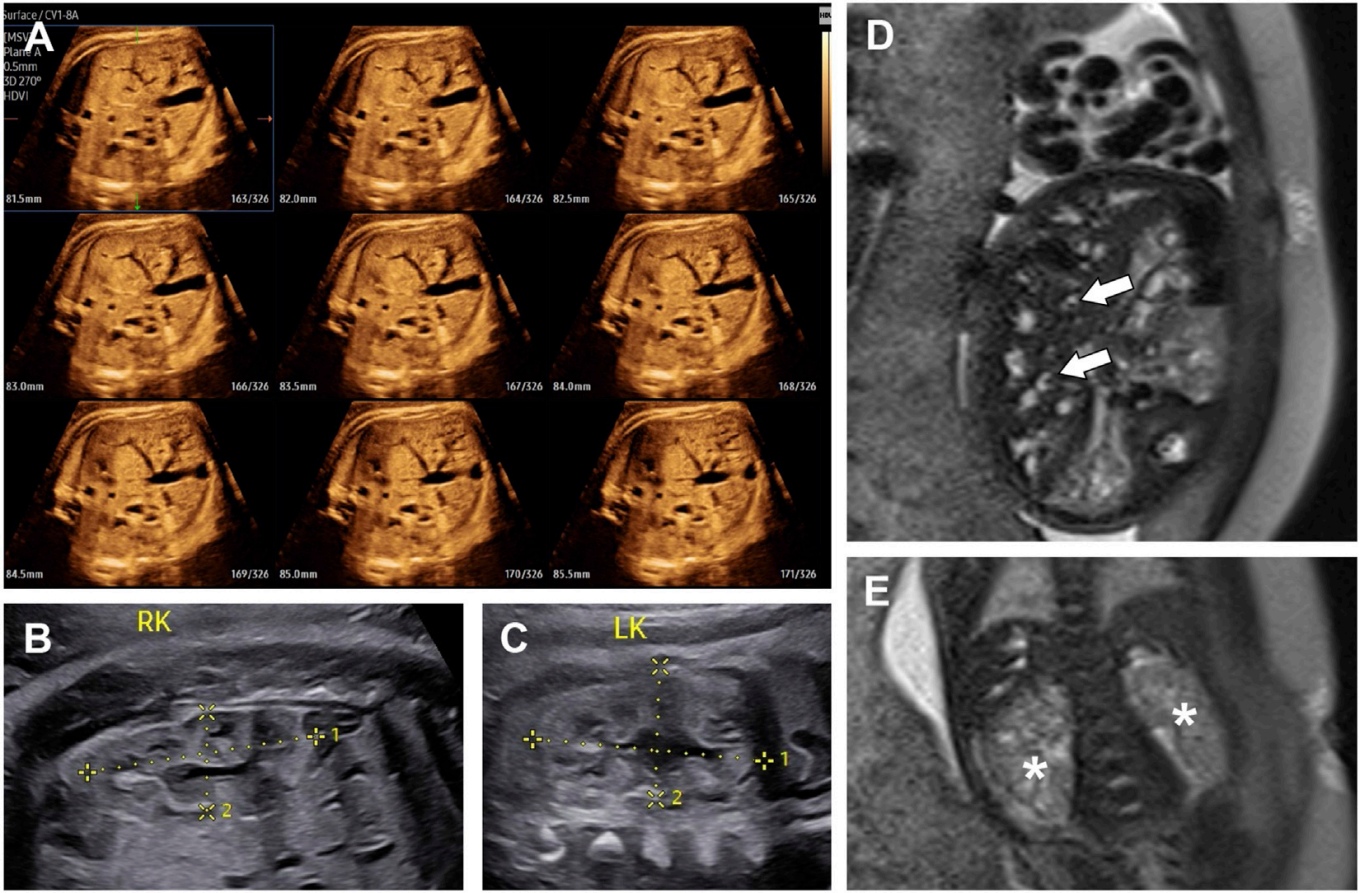
The ultrasound images **(A–C)** and T2-weighted MRI images **(D,E)** of fetal abdomen at 33+4 weeks of gestation. **(A)** Multi-Slice view of fetal liver, showing multiple small tubular and cystic anechoic structures. **(B)** Right kidney (4.06 × 1.75 cm). **(C)** left kidney (3.88 × 2.22 cm). Both kidneys showed no obvious enlargement and loss of corticomedullary differentiation. **(D)** The heterogeneous liver with multiple dilated bile ducts. The central dot sign was indicated by arrows. **(E)** Two slightly enlarged kidneys (asterisks), showing numerous tiny cysts and no corticomedullary differentiation.

A 39-year-old woman was referred to our hospital for ultrasound consultation due to fetal hepatic anomalies detected during a routine sonographic examination. This was her second pregnancy. Her first pregnancy had ended in an induced abortion. A detailed fetal ultrasound examination at 33+4 weeks of gestation revealed multiple small tubular and cystic dilatations in the liver (Figure 1A). Both kidneys were echogenic and showed no corticomedullary differentiation (Figures 1B,C). No other abnormalities were detected.

On the same day, T2-weighted magnetic resonance imaging (MRI) was performed. The dilated biliary ducts appeared as tubular or cystic hyperintense lesions on T2-weighted sequences, with the “central dot” (C-DOT) sign observed (Figure 1D). Both kidneys were slightly enlarged, with loss of corticomedullary differentiation and multiple tiny cystic lesions with hypersignal on the T2-weighted sequence (Figure 1E). After clinical counseling, the couple opted to terminate the pregnancy. As the parents declined autopsy, we were unable to confirm the presence of CHF in the fetus, and thus could not definitively distinguish between CD and CS. As a result, a diagnosis of CD/CS with ARPKD was suggested.

#### 3.1.2 Family 2

**FIGURE 2:**
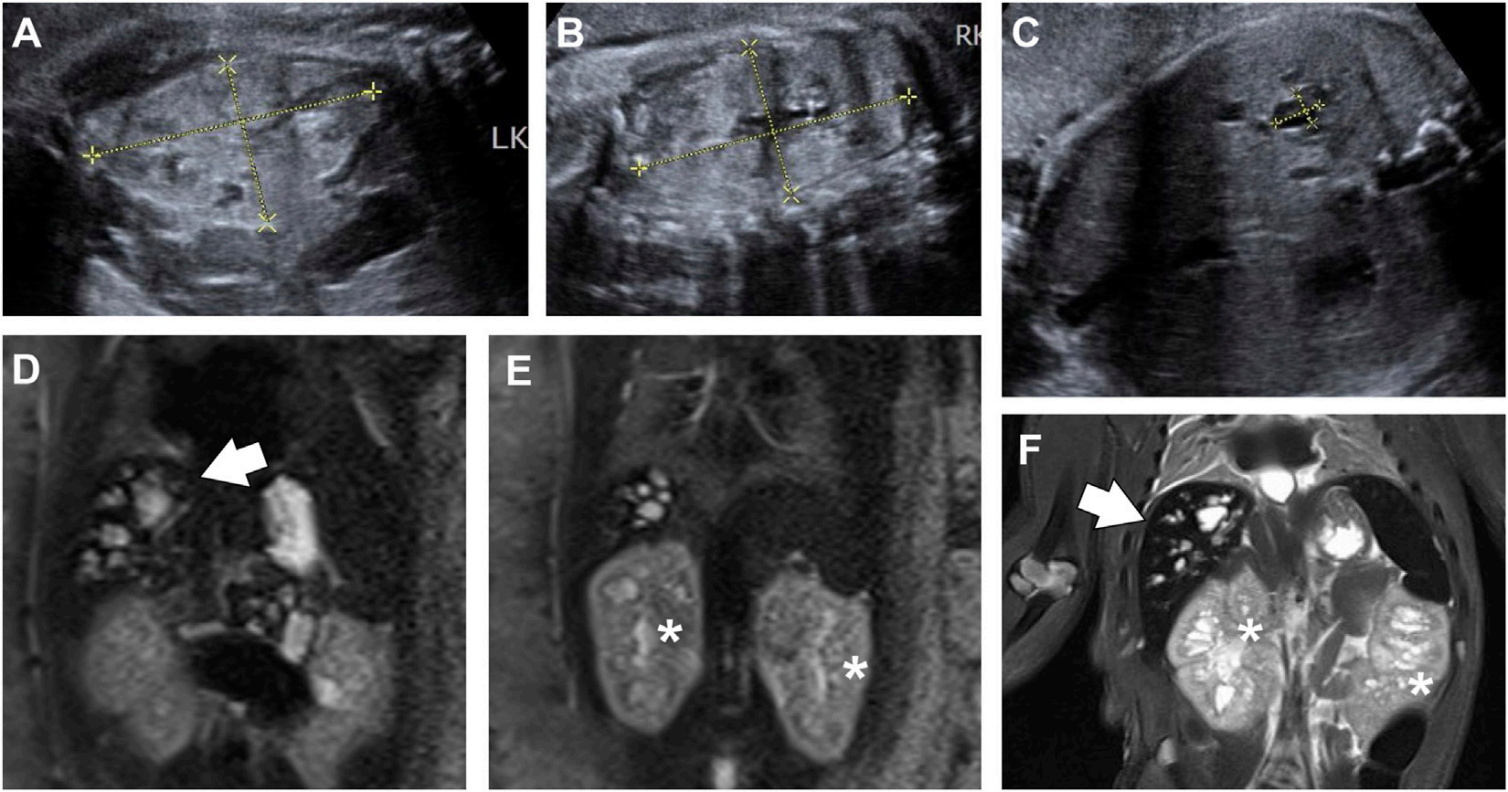
View of fetal abdomen before **(A–E)** and after birth **(F)**. **(A-C)** Ultrasound images of fetal kidney **(A,B)** and liver **(C)** at 39+2 weeks of gestation. Both kidneys (**(A,B)**; left, 6.28 cm × 3.57 cm; right, 6.67 cm × 3.65 cm) were enlarged and echogenic, with no corticomedullary differentiation. The right lobe of the liver **(C)** showed diffuse cystic structures, one of which was 1.03 cm × 0.74 cm in size. **(D,E)** T2-weighted MRI image of fetal abdomen at 39+6 weeks. The fetal liver (D, arrows) was hyperintense on T2-weighted sequences, showing multiple cystic and tubular structures. Both kidneys [**(E)**, asterisks] were enlarged and heterogeneous, with numerous micro cysts. **(F)** T2-weighted MRI image of the baby, showing dilated intrahepatic biliary ducts (arrows), and slightly enlarged kidneys (asterisks) with cystic structures.

A 26-year-old primigravida presented to our hospital for ultrasound consultation due to increased echogenicity in the fetal kidneys and oligohydramnios at 38 weeks of gestation. At 39+2 weeks of gestation, ultrasound examination revealed several anechoic cystic lesions in the right lobe of the fetal liver (Figure 2C). Color Doppler did not show flow within the lesions. Both kidneys were enlarged and echogenic, with no corticomedullary differentiation (Figures 2A,B). Oligohydramnios was noted, with an amniotic fluid depth of 2.3 cm and an amniotic fluid index of 6.1 cm.

At 39+6 weeks, an MRI study was performed. T2-weighted sequences revealed multiple cystic and tubular dilatations of varying sizes within the hepatic parenchyma (Figure 2D). Both kidneys were enlarged and exhibited numerous cystic structures that appeared hyperintense on the T2 sequence (Figure 2E). The baby was delivered vaginally at term but died a few days later. Postnatal T2-weighted MRI confirmed the presence of similar findings in the kidneys and liver (Figure 2F). The couple refused autopsy, so pathological confirmation of CHF was not obtained. Based on these findings, a diagnosis of CD/CS with ARPKD was suggested.

### 3.2 Genetic analysis

#### 3.2.1 Family 1

**FIGURE 3:**
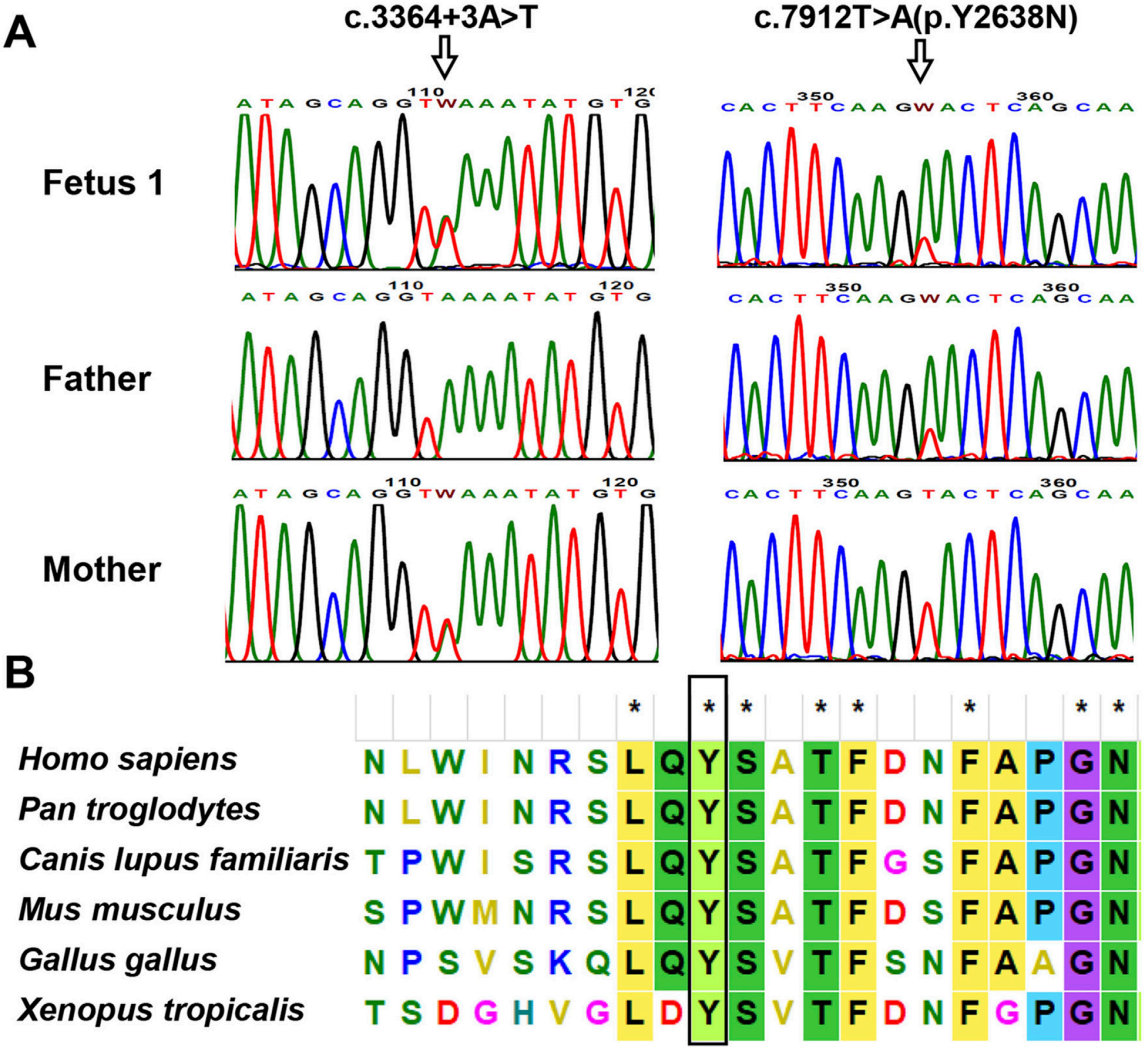
Analysis of the identified *PKHD1* variants in family 1. **(A)** Sanger sequencing chromatogram of *PKHD1* variants. The variant c.3364 + 3A>T in *PKHD1* was detected in Fetus 1 and the mother; the c.7912T>A was identified in fetus 1 and the father. The mutated sites were marked by arrows. **(B)** Multiple sequence alignment of *PKHD1* from different species. The tyrosine residue at position 2638, marked by the black rectangle, was highly conserved in vertebrates.

Trio WES identified two heterozygous variants, c.7912T>A and c.3364 + 3A>T, in the *PKHD1* gene (NM_138694.3) in fetus 1. The father was a heterozygous carrier of c.7912T>A, and the mother was a heterozygous carrier of c.3364 + 3A>T. Both variants were confirmed in this family by Sanger sequencing (Figure 3A). The c.7912T>A variant, located in exon 50 of the *PKHD1* gene, caused a substitution of tyrosine to asparagine at amino acid position 2638. Although both residues are polar, substitution of tyrosine (which has an aromatic ring) with asparagine (which does not) leads to a loss of aromaticity. This variant was very rare in the normal population database (Table 1). Multiple bioinformatic tools predicted it to be damaging or disease-causing (Table 1), and sequence alignment analysis revealed it to be highly conserved among vertebrates (Figure 3B). Additionally, this variant has been recorded in the ClinVar database with conflicting classifications of pathogenicity and in the HGMD as a disease-causing mutation. The c.3364 + 3A>T variant was absent from the normal population database and not recorded in ClinVar or HGMD. In silico tools, including SpliceAI, dbscSNV, varSEAK, and MaxEntScan, predicted that the c.3364 + 3A>T intronic change, located near the donor site of intron 29, would likely affect splicing and disturb the function of the authentic donor site, causing exon skipping (Table 1). According to the ACMG/AMP guidelines, the variant c.7912T>A and c.3364 + 3A>T were both classified as variants of uncertain significance (VUS) (PM2_Supporting + PP3).

**TABLE 1:** *PKHD1* candidate variants identified in this study and previous studies, along with their bioinformatic functional predictions and allele frequencies in the normal population.

Fetus	Gene	Exon/Intron	Nucleotide change	Normal population database				In silico prediction software						Splicing prediction algorithm			
				1000G	ESP6500	ExAC	GnomAD	SIFT	Polyphen2	MutationTaster	Provean	REVEL	CADD	SpliceAI	dbscSNV	varSEAK	MaxEntScan
Fetus 1	*PKHD1*	50	c.7912T>A p.Tyr2638Asn	—	—	0.000017	0.000016	0 (damaging)	0.982 (Probably Damaging)	0.998 (Disease causing)	−7.810 (Damaging)	0.806 (Damaging)	30.0 (Damaging)	—	—	—	—
	*PKHD1*	29	c.3364 + 3A>T	—	—	—	—	—	—	—	—	—	—	donor loss 0.85	ADA:0.9999; RF: 0.958 (Splice-altering)	−76.20% (Exon Skipping)	70.4% (Splice-altering)
Fetus 2	*PKHD1*	59	c.9901G>T p.E3301X	—	—	0.0000083	—	—	—	1 (Disease causing)	—	—	—	—	—	—	—
	*PKHD1*	24	c.2507T>C p.V836A	0.0002	—	0.000091	0.000032	0 (damaging)	0.861 (Possibly_damaging)	0.677 (Polymorphism)	−2.77 (Damaging)	0.841 (Damaging)	24.9 (Damaging)	—	—	—	—
Sgro et al. (2004)	*PKHD1*	55	IVS55 + 1G → A (c.8642 + 1G>A)	—	—	—	0.000001987	—	—	1 (disease causing)	—	—	—	Acceptor Loss, 0.95; Donor Loss, 1	ADA:1; RF:0.934 (Splice-atering)	76.68%(Exon Skipping)	79.57%(Splice-altering)
	*PKHD1*	50	c.8068T>C(p.W2690R)	—	—	—	0.00003098	0 (damaging)	0.997 (Probably Damaging)	1 (disease causing)	−11.82(Deleterious)	0.849 (Deleterious)	27.7 (Damaging)	—	—	—	—
Rivas et al. (2019)	*PKHD1*	53	c.8407T>C(p.C2803R)	0.000199681	—	0.000008241	0.00001487	0 (damaging)	0.999 (Probably Damaging)	0.98 (disease causing)	−5.25(Deleterious)	0.854 (Deleterious)	25.9 (Damaging)	—	—	—	—

#### 3.2.2 Family 2

**FIGURE 4:**
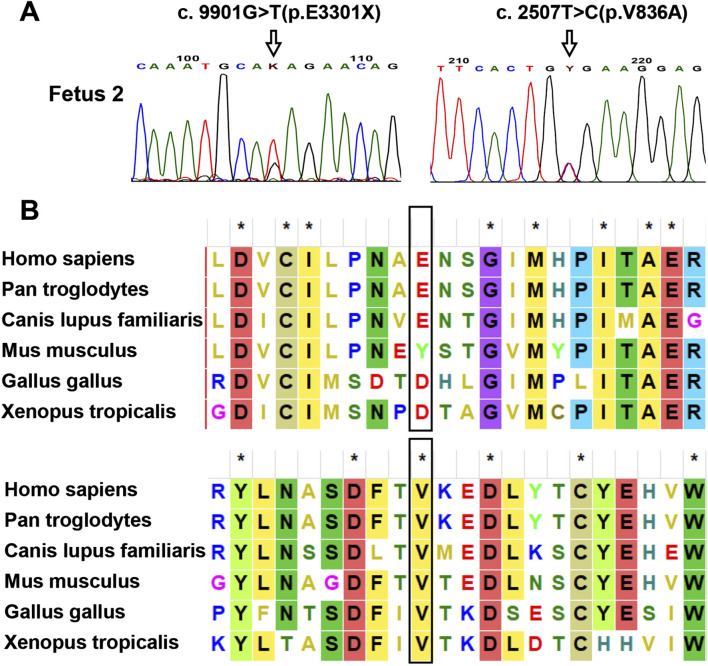
Analysis of the identified *PKHD1* variants in fetus 2. **(A)** Sanger sequencing verification of the *PKHD1* variants. The mutated sites were marked by arrows. **(B)** Sequence alignment of the *PKHD1* protein among different species. The Val836 residue of *PKHD1* was conserved in vertebrates, while the Glu3301 residue was not. The mutated sites were marked by black rectangles.

As no peripheral venous blood samples were obtained from the parents, only WES on the fetus was performed. Based on the WES data, two *PKHD1* variants, c.9901G>T (p.Glu3301Ter) and c.2507T>C (p.Val836Ala), were identified as the most likely to be associated with the phenotype in fetus 2. These variants were verified by Sanger sequencing (Figure 4A). No other variants in genes related to ARPKD or Caroli disease/syndrome were detected. The frequencies of these two variants in normal population databases and functional predictions from bioinformatic software are detailed in Table 1. Multiple sequence alignment analysis for these two variants revealed that the Val836 residue was highly conserved across a range of different species (Figure 4B). Both variants are documented in the ClinVar database as Pathogenic/Likely pathogenic and in the HGMD as disease-causing mutations According to the ACMG/AMP guidelines, the c.2507T>C (p.Val836Ala) variant was classified as VUS (PM2_Supporting + PP3), and the c.9901G>T (p.Glu3301Ter) variant was classified as Likely Pathogenic (PVS1+PM2_Supporting).

### 3.3 Splicing study of the *PKHD1* c.3364 + 3A>T variant by minigene assays

**FIGURE 5:**
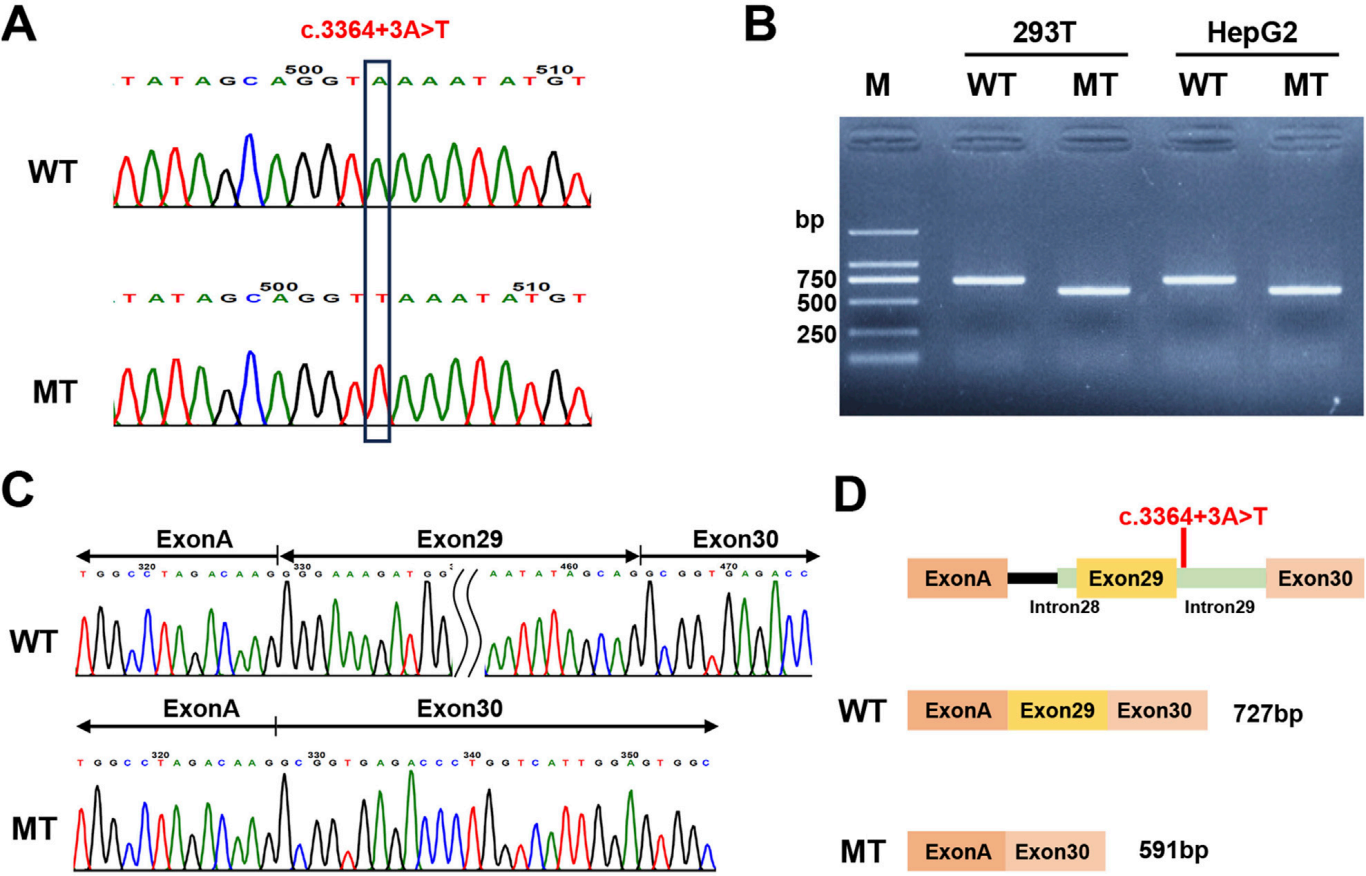
Minigene assay analysis of the *PKHD1* c.3364 + 3A>T with the pcMINI-C vector. **(A)** Verification of the constructed pcMINI-C-*PKHD1*-WT/MT recombinant vectors by Sanger sequencing. **(B)** The agarose gel electrophoresis results of the RT-PCR products in 293T and HepG2 cell lines. MT showed a lower band than WT. **(C)** The corresponding Sanger sequencing results of the RT-PCR products. The MT showed exon 29 skipping. **(D)** Schematic diagram of the vector construction and the alternative splicing events in the minigene assay.

Based on *in silico* predictions suggesting that the c.3364 + 3A>T variant may affect *PKHD1* RNA splicing, we conducted a minigene assay using pcMINI and pcMINI-C vectors. After transfecting HepG2 and HEK293T cells, we examined the minigene-derived transcripts using RT-PCR. Electrophoretic analysis demonstrated that the wild-type constructs (pcMINI-*PKHD1*-WT and pcMINI-C-*PKHD1*-WT) produced a band of larger size than the corresponding mutant constructs (pcMINI-*PKHD1*-MT and pcMINI-C-*PKHD1*-MT) in both HepG2 and HEK293T cell lines (Figure 5B; Supplementary Figure S1B). Sanger sequencing of these products confirmed a canonical splicing pattern in the wild-type constructs, whereas the mutant constructs exhibited aberrant splicing with skipping of exon 29 (Figure 5C; Supplementary Figure S1C). This exon skipping would lead to a premature stop codon (c.3229_3364del, p.(Gly1077Alafs*12)) in the FPC protein. Schematic diagrams illustrating the splicing patterns in both constructs are shown in Figure 5D; Supplementary Figure S1D.

### 3.4 Clinical and genetic characteristics of the patients with prenatally diagnosed CD or CS

**FIGURE 6:**
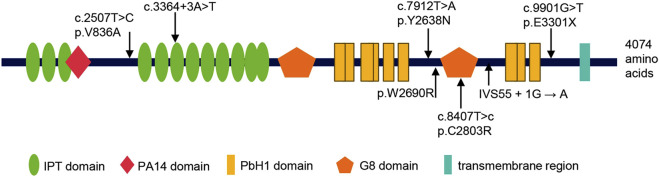
Summary of all *PKHD1* variants identified in fetuses with CD or CS. Above: the variants identified in this study; below: the variants reported in previous studies. The protein Fibrocystin/Polyductin (FPC), encoded by *PKHD1*, is annotated with its functional domains: IPT/TIG domain (Ig-like, plexins and transcription factors), PA14 domain (Pfam Entry 14), PbH1 domain (parallel beta-helix repeats), G8 domain (eight conserved glycine residues), and transmembrane region.

A comprehensive review of the literature revealed that six cases with prenatally diagnosed CD or CS have been reported in the literature, only two of which underwent genetic testing (Supplementary Table S4). With the inclusion of the four variants identified in the present study, a total of seven *PKHD1* variants have now been described (Table 1; Figure 6). These include one nonsense, two splicing, and four missense variants. All patients carrying these mutations were also affected by ARPKD. From Figure 6, we found that all the missense variants lie outside known functional domains of fibrocystin. However, multiple sequence alignment analysis revealed that they are highly conserved across vertebrates (Supplementary Figure S2), and *in silico* tools predicted them to be damaging or disease-causing (Table 1). Notably, three of the identified missense variants were located within the amino acid region 2625–4074 of FPC. This region has previously been associated with poorer hepatic outcomes in pediatric ARPKD patients (Burgmaier et al., 2021).

## 4 Discussion

CD or CS is a rare inherited disorder characterized by segmental dilatation of large intrahepatic bile ducts (Caroli et al., 1958; Castro et al., 2017), which is primarily caused by pathogenic variants in the *PKHD1* gene (Courcet et al., 2015; Kyalwazi et al., 2025). In this study, we report two Chinese fetuses with CD/CS at 33 and 39 weeks of gestation, respectively. To identify the genetic bases of these cases, WES was performed and revealed *PKHD1* variants associated with the phenotypes. Furthermore, a minigene assay confirmed that the variant c.3364 + 3A>T affected splicing. To our knowledge, prenatal diagnosis of CD or CS is very rare, with only two previously reported cases undergoing genetic analysis and showing *PKHD1* mutations (Hussman et al., 1991; Yuksel et al., 2002; Sgro et al., 2004; Castro et al., 2017; Rivas et al., 2019; Castro et al., 2020). Our study adds to the limited literature on prenatally diagnosed CD or CS patients and expands the spectrum of *PKHD1* variations in these patients.

*PKHD1* was first reported as the causative gene of ARPKD, and later found to be responsible for CD or CS. In several Pkhd1 mouse models, random mutagenesis or targeted genetic engineering of the Pkhd1 gene induced liver phenotypes resembling human disease, including dilated bile ducts, hepatic fibrosis and other ductal plate malformations (Yang et al., 2023). Further studies demonstrated that the FPC protein, encoded by *PKHD1*, is primarily localized to the renal collecting ducts and hepatic bile ducts (Swanson, 2021). Although the exact function of FPC protein remains unclear, it is hypothesized to act as a receptor, and may participate in the regulation of ductal cell formation, proliferation, apoptosis, adhesion, and signal transduction (Goggolidou and Richards, 2022). In humans, pathogenic variants in *PKHD1* primarily cause ARPKD, and are rarely reported in patients with CD or CS. The documented types of *PKHD1* variants in CD or CS are diverse, including frameshift, nonsense, missense, splice-site variants, and other types (Hao et al., 2014; Courcet et al., 2015; Mi et al., 2017; Giacobbe et al., 2022).

In fetus 1, dilated intrahepatic bile ducts and ARPKD were observed. Two compound heterozygous *PKHD1* variants, c.7912T>A (p.Tyr2638Asn) and c.3364 + 3A>T, were identified, consistent with the autosomal recessive inheritance pattern of CD or CS. The c.7912T>A variant was identified in a fetus at 29 weeks of gestation with ARPKD and hepatic portal fibrosis (Denamur et al., 2010). Although both residues are polar, the substitution of tyrosine to asparagine introduces large physicochemical differences: loss of aromaticity and reduced side chain volume (ClinVar/Labcorp: SCV002127856.2). Multiple sequence alignment analysis revealed that this residue is highly conserved among vertebrates. The splice site variant c.3364 + 3A>T has also been previously reported in a 6-month-old Chinese child with sponge kidney and dilated intrahepatic bile ducts (Qiu et al., 2020). However, no functional study has been conducted. In this study, multiple splicing prediction tools indicated that it is likely to affect splicing. Since minigene assays have been demonstrated to yield splicing results with nearly 100% similarity to endogenous transcripts (van der Klift et al., 2015), we performed minigene experiments with pcMINI and pcMINI-C vectors. The results showed that the splice site variant c.3364 + 3A>T caused exon 29 skipping, which may result in a frameshift and generation of a premature stop codon (p.Gly1077Alafs*12) in FPC protein. This premature stop codon is likely to result in loss-of-function of the protein either by a truncation or by nonsense-mediated decay of the *PKHD1* mRNA (Ebner et al., 2017). As a result, the variant c.3364 + 3A>T was reclassified from VUS to likely pathogenic variants (PVS1 + PM2_Supporting), based on the ACMG/AMP guidelines.

In Fetus 2, bioinformatic analyses of the annotated sequencing data identified only two *PKHD1* variants, c.9901G>T (p.Glu3301Ter) and c.2507T>C (p.Val836Ala), as the variants mostly associated with the CD and ARPKD phenotype. The variant c.9901G>T was classified as likely pathogenic (PVS1 + PM2_Supporting) according to the ACMG/AMP guidelines. This variant has been previously reported in a Chinese fetus at 20 weeks of gestation with ARPKD (Guo et al., 2012). The other variant, c.2507T>C (p.Val836Ala), was classified as a VUS (PM2_Supporting + PP3) by ACMG/AMP guidelines. It has been reported in several cases with liver or kidney lesions, especially CD and/or ARPKD (Hao et al., 2014; Liu et al., 2014; Kang et al., 2016; Xiying et al., 2018; Qiu et al., 2020; Wang et al., 2021; Ishiko et al., 2022). Qiu et al. suggested that it may be a hot-spot mutation associated with hepatic lesions in Chinese children with ARPKD (Qiu et al., 2020). Although parental samples were unavailable for verification of these variants, the absence of liver or kidney anomalies in the parents suggests a recessive inheritance pattern for the phenotype observed in fetus 2. Collectively, these findings strongly suggest that the heterozygous variants in *PKHD1* are likely the cause of the condition in fetus 2.

In this work, the missense variant c.7912T>A(p.Tyr2638Asn) and the nonsense variant c.9901G>T (p.Glu3301Ter), were found in CD/CS patients for the first time. This novel observation may reflect the extensive phenotypic variability associated with pathogenic *PKHD1* variants. It has been reported that different mutation types and combinations of *PKHD1* variants can lead to varying clinical presentations, ranging from asymptomatic patients to those with severe liver and kidney involvement (Giacobbe et al., 2022). For *PKHD1* variants in patients with ARPKD, genotype-phenotype correlations regarding variant type and location have been reported. However, no such correlations have been established for CD or CS patients harboring *PKHD1* variants to date. To date, only seven *PKHD1* variants (including four variants in our study) have been described in cases with prenatally diagnosed CD or CS (Figure 6), 57% of which are missense variants. We found that the missense variants are not located in the known functional domains of FPC (Figure 6). However, they are highly conserved across vertebrates, and predicted to be deleterious by multiple *in silico* tools. Three of the four missense variants, including c.7912T>A(p.Y2638N), p.W2690R, and c.8407T>C(p.C2803R), are clustered within the amino acid region 2625–4074 of FPC, and the patients carrying these variants were also presented with ARPKD (Sgro et al., 2004; Rivas et al., 2019). To our knowledge, CD and CS are often associated with severe hepatobiliary complications over time, including recurrent cholangitis, portal hypertension, and even cholangiocarcinoma. These findings may provide additional support for the previous observation that the missense variants in the amino acid region 2625–4074 were associated in pediatric ARPKD patients with poorer hepatic outcomes and an elevated risk of severe liver-related complications (Burgmaier et al., 2021). Moreover, Yang et al. reported that the *PKHD1*cyli/cyli(cyli) mice, harboring an indel mutation c.7588_7589delGGinsT (p.G2530VfsTer15) in *PKHD1*, developed a severe liver pathology (Yang et al., 2023). Fetus 2 in our study carried a compound heterozygous variants in *PKHD1*, a nonsense variant c.9901G>T(p.Glu3301Ter) and a missense variant c.2507T>C(p.Val836Ala), which is located in the N-terminal amino acid region 709–1837 of FPC. Two missense variants in this region or a missense variant in this region and a null variant have previously been associated with a lower frequency of chronic kidney failure in postnatal ARPKD patients (Burgmaier et al., 2021). Besides, postnatal cases with the p.Val836Ala variant also predominantly presented with CD or CS and a mild renal involvement (Hao et al., 2014; Liu et al., 2014; Qiu et al., 2020; Wang et al., 2021). In contrast to these observations, Fetus 2 exhibited severe prenatal kidney disease at 39 weeks of gestation, including enlarged kidneys, loss of corticomedullary differentiation, oligohydramnios, and then early postnatal death. We hypothesize that this discrepancy may be attributed to the inclusion criteria of Burgmaier et al.’s study, which excluded fetus with terminated pregnancy or neonates that died in the first month of life. Indeed, missense variants within the 709–1837 amino acid region have been reported in ARPKD cases with medical pregnancy termination or early postnatal demise (Denamur et al., 2010). These observations underscore the complexity of genotype–phenotype correlations in ARPKD patients with *PKHD1* variants and emphasize the necessity of including prenatal cases and early-lethal cases to refine genotype-phenotype models for this disease spectrum.

Most cases of CD and CS are diagnosed after birth, while prenatally diagnosed cases are very limited. To our knowledge, only 8 cases (including the two cases in this study) have been reported (Supplementary Table S5) (Hussman et al., 1991; Yuksel et al., 2002; Sgro et al., 2004; Castro et al., 2017; Rivas et al., 2019; Castro et al., 2020). Prenatal diagnosis of CD or CS by ultrasound remains challenging, as the sonographic differential diagnosis of entities that present as cystic dilatations in and about the liver is wide, such as polycystic liver disease, solitary nonparasitic cyst, mesenchymal hamartoma, and Caroli’s disease (Hussman et al., 1991). Furthermore, prenatal ultrasound often fails to detect the “central dot” sign—a key differentiating feature of CD and CS caused by a portal vein branch protruding into the bile duct (Rivas et al., 2019). In such cases, MRI serves as a valuable supplementary diagnostic tool due to its enhanced capability to visualize the dilated biliary tree and the “central dot” sign (Castro et al., 2020). Previous studies have utilized a combined ultrasound-MRI approach for the prenatal diagnosis of CD or CS (Castro et al., 2017; Rivas et al., 2019; Castro et al., 2020). In the present study, we also employed this combined method, further confirming its feasibility for the prenatal assessment of CD or CS. However, consistent with previous studies, we found that the central dot sign is not always detectable, even by MRI. Furthermore, all of the cases were found after 28 weeks of gestation, except two before 24 weeks of gestation. These discrepancies may be related to the intensity of ductal dilatation or the limited capability of ultrasound and MRI to detect this small characteristic prenatally (Castro et al., 2020). As shown in Supplementary Table S4, the prognosis of the prenatally diagnosed CD or CS is very poor. Of the eight reported fetuses, five died (one *in utero* and four after birth), one was terminated, and only two survived after birth with one undergoing renal and liver transplantation. This poor prognosis highlights the importance of early diagnosis and intervention. In recent years, with the advancement of high-throughput sequencing technology, WES has become a powerful tool for investigating the genetic basis of human diseases. Given the challenges of prenatal diagnosis for CD or CS, the poor prognosis and the typical detection after the second trimester, we strongly recommended performing WES in the probands of affected families to identify pathogenic *PKHD1* variants. This strategy not only allows for the assessment of genetic risks, but also facilitates early prenatal molecular diagnosis in subsequent pregnancies, thereby providing more time for family decision-making.

In this study, we reported two rare cases of prenatally diagnosed CD/CS and identify the associated *PKHD1* variants. This represents the first report of prenatal diagnosis of CD or CS along with comprehensive genetic analysis in Chinese cohort. Our findings expand the *PKHD1* mutation spectrum in CD or CS patients and confirms the pathogenicity of the splice site variant c.3364 + 3A>T through minigene assays. We also emphasize the necessity of including prenatal cases and early-lethal cases to refine the reported genotype-phenotype correlations in ARPKD patients with *PKHD1* variants. Given the poor prognosis of prenatally diagnosed Caroli disease and its typical detection after the second trimester, we recommend performing WES in the proband of affected families, which could facilitate timely genetic counseling and enables early intervention strategies in subsequent pregnancies. Future studies should focus on larger cohorts and functional analyses to further elucidate the genotype-phenotype correlations and improve clinical management.

## Data Availability

The datasets presented in this article are not readily available because of ethical/privacy restrictions. Requests to access the datasets should be directed to the corresponding author.
