# Supplementary material for: Prenatal diagnosis and molecular characterization of PKHD1 variants in two Chinese fetuses with Caroli disease/syndrome
Source: Front Genet. 2025 Sep 30;16:1651306. doi: 10.3389/fgene.2025.1651306 (PMC12517586; doi:10.3389/fgene.2025.1651306)
Supplement: Supplementary file 1 [file DataSheet1.docx]

Supplementary Material

# Supplementary Figures





**Supplementary Figure S1.** Minigene splicing analysis of the *PKHD1* c.3364+3A>T with the pcMINI vector. **(A)** Sanger sequencing results of the constructed pcMINI-PKHD1-wt/mt vectors. **(B)** Agarose gel electrophoresis of the RT-PCR products in 293T and HepG2 cell lines. MT showed a lower band than WT. **(C)** Sanger sequencing of the RT-PCR products. The MT showed exon 29 skipping. **(D)** The schematic representation of the vector construction and the alternative splicing events in the minigene assay.


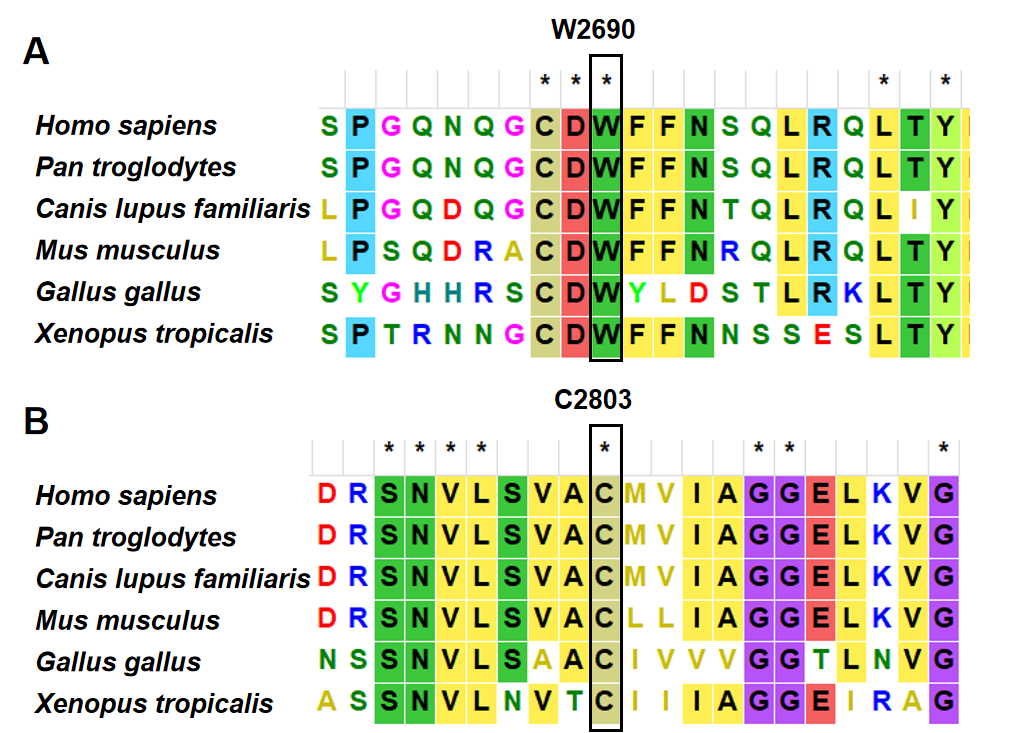


**Supplementary Figure S2.** Multiple sequence alignment of PKHD1 from different species. The Tryptophan residue at position 2690 **(A)** and the Cysteine residue at position 2803 **(B)** were highly conserved in vertebrates. Both residues were marked by black rectangle.

# Supplementary Tables

**Supplementary Table S1.** Primer sequences used in verification of the *PKHD1* mutations in Family 1 and Fetus 2.

| Patient | Primer Name | Primer sequence (5´‐3´) |
| --- | --- | --- |
| Family 1 | PKHD1-c.3364+3-F | GGGAAAGATGGACGCATTGTG |
|  | PKHD1-c.3364+3-R | TTCCCACATCCTTTTCATGATGG |
|  | PKHD1-c.7912-F | TCGCTTCAAGTCGTGGGAAA |
|  | PKHD1-c.7912-R | GAGGATGTCAGGGTAAGGCG |
| Fetus 2 | PKHD1-c.9901-F | TCGATATTTGTGGCTGGTGGT |
|  | PKHD1-c.9901-R | ACCTGGGTTGTAATGAAGGAAAGT |
|  | PKHD1-c.2507-F | GGCTGGGGATTCTACTGAAGTT |
|  | PKHD1-c.2507-R | GCAGCAAATCCATGCCACTA |

**Supplementary Table S2.** Primer sequences used in the minigene assay with the pcMINI-C vector.

| **Process** | **Name of Primer** | **Primer sequence (5’→3’)** | **Detailed Description** |
| --- | --- | --- | --- |
| Step 1 of the NEST-PCR | 50651-PKHD1-F | ctggcagaaccgggtagtat | Amplification of the wild-type genomic fragment of *PKHD1* (named pcMINI-C-1) from the father’s genomic DNA by NEST-PCR. |
|  | 52800-PKHD1-R | gtcacctggggcagcaaatt |  |
| Step 2 of the NEST-PCR  （pcMINI-C-1） | 50933-PKHD1-F | tctccatgttggtcaggttg |  |
|  | 52573-PKHD1-R | gggaagttacctgctactct |  |
| Amplification of the pcMINI-C-2 | 55937-PKHD1-F | gaccgttagctctgcaaact | Amplification of the wild-type genomic fragment of *PKHD1* (named pcMINI-C-2) from the father’s genomic DNA by PCR. |
|  | 57016-PKHD1-R | ctgtgaggtactggatgtgg |  |
| Amplify the left half of pcMINI‐C-PKHD1-WT | pcMINI-C-PKHD1-KpnI-F | ggtaGGTACCtttcttgagtagtcactgtt | The nested PCR product (pcMINI-C-1) was used as the template, and the left half (1145bp) of wild‐type fragment (pcMINI‐C-PKHD1-WT) was amplified by PCR. |
|  | pcMINI-C-PKHD1-linker-R | cagcctcccaagcagttaactacaagtaga |  |
| Amplify the right half of pcMINI‐C-PKHD1-WT and then the fragment pcMINI‐C-PKHD1-WT | pcMINI-C-PKHD1-linker-F | tctacttgtagttaactgcttgggaggctg | The PCR product (pcMINI-C-2) was used as the template, and the right half (513bp) of wild‐type fragment (pcMINI‐C-PKHD1-WT) was amplified by PCR.  The 1:1 mixture of left and right half of wild‐type fragment was used as the template, and the pcMINI-C-PKHD1-KpnI-F and pcMINI-C-PKHD1-BamHI-R was used as primers to amplify the 1628bp wild‐type fragment (pcMINI‐C-PKHD1-WT). |
|  | pcMINI-C-PKHD1-BamHI-R | TAGTGGATCCcCCTTGTGAGTGAATGCTGAC |  |
| Amplify the left half of pcMINI-C-PKHD1-MT | pcMINI-C-PKHD1-KpnI-F | ggtaGGTACCtttcttgagtagtcactgtt | The clone vector including 1628bp wild-type fragment was used as the template, and the left half (682bp) of mutant‐type fragment (pcMINI‐C-PKHD1-MT) was amplified by PCR. |
|  | PKHD1-mut-R | cagtatcacatatttAacCTGCTATATTGCT |  |
| Amplify the right half of pcMINI-C-PKHD1 MT and then the fragment of pcMINI-C-PKHD1-MT | PKHD1-mut-F | AGCAATATAGCAGgttaaatatgtgatactg | The clone vector containing 1628bp wild‐type fragment was used as the template, and the right half (977bp) of mutant‐type fragment (pcMINI‐C-PKHD1-MT) was amplified by PCR.  The 1:1 mixture of left and right half of mutant‐type fragment was used as the template, and the pcMINI-C-PKHD1-KpnI-F and pcMINI-C-PKHD1-BamHI-R was used as primers to amplify the 1628bp mutant‐type fragment (pcMINI‐C-PKHD1-MT). |
|  | pcMINI-C-PKHD1-BamHI-R | TAGTGGATCCcccttgtgagtgaatgctgac |  |
| Amplify the pcMINI-C-PKHD1 from RT-PCR products | pcMINI-C-F | ctagagaacccactgcttac | After the DNA fragment of pcMINI-C-PKHD1-WT and the fragment of pcMINI-C-PKHD1-MT were ligated to the pcMINI-C vector and transfected into HEK293T and HepG2 cells for 48 hours, total RNA was extracted and the RT-PCR products were verified by PCR. |
|  | pcMINI-C-R | tagaaggcacagtcgagg |  |

**Supplementary Table S3.** Primer sequences used in the minigene assay with the pcMINI vector.

| **Process** | **Name of Prime** | **Primer sequence (5’→3’)** | **Detailed Description** |
| --- | --- | --- | --- |
| Step 1 of the NEST-PCR  （pcMINI） | 50651-PKHD1-F | ctggcagaaccgggtagtat | Amplification of the wild-type genomic fragment of *PKHD1* (named pcMINI) from the father’s genomic DNA by NEST-PCR. |
|  | 52800-PKHD1-R | gtcacctggggcagcaaatt |  |
| Step 2 of the NEST-PCR  （pcMINI） | 50933-PKHD1-F | tctccatgttggtcaggttg |  |
|  | 52573-PKHD1-R | gggaagttacctgctactct |  |
| Amplify the pcMINI- PKHD1-WT | pcMINI-PKHD1-KpnI-F | ggtaGGTACCttcttatttaccccttacag | The nested PCR product (pcMINI) was used as the template, and the 900bp wild‐type fragment (pcMINI‐PKHD1-WT) was amplified by PCR. |
|  | pcMINI-PKHD1-BamHI-R | TAGTGGATCCttaactacaagtagataagt |  |
| Amplify the left half of pcMINI-PKHD1-MT | pcMINI-PKHD1-KpnI-F | ggtaGGTACCttcttatttaccccttacag | The nested PCR product (pcMINI) was used as the template, and the left half (442bp) of mutant‐type fragment (pcMINI‐PKHD1-MT) was amplified by PCR. |
|  | PKHD1-mut-R | cagtatcacatatttAacCTGCTATATTGCT |  |
| Amplify the right half of pcMINI-PKHD1-MT and then the fragment of pcMINI-PKHD1-MT | PKHD1-mut-F | AGCAATATAGCAGgtTaaatatgtgatactg | The nested PCR product (pcMINI) was used as the template, and the right half (489bp) of mutant‐type fragment (pcMINI‐PKHD1-MT) was amplified by PCR.  The 1:1 mixture of left and right half of mutant‐type fragment was used as the template, and the pcMINI-PKHD1-KpnI-F and pcMINI-PKHD1-BamHI-R was used as primers to amplify the 900bp mutant‐type fragment (pcMINI‐PKHD1-MT). |
|  | pcMINI-PKHD1-BamHI-R | TAGTGGATCCttaactacaagtagataagt |  |
| Amplify the pcMINI -PKHD1 | pcMINI-F | tagaaggcacagtcgagg | After the DNA fragment of pcMINI-PKHD1-WT and the fragment of pcMINI-PKHD1-MT were ligated to the pcMINI vector and transfected into HEK293T and HepG2 cells for 48 hours, total RNA was extracted and the RT-PCR products were verified by PCR. |
|  | pcMINI-R | ctagagaacccactgcttac |  |

**Supplementary Table S4.** List of cases of Caroli’s disease or Caroli’s syndrome diagnosed prenatally in literature.

| **No.** | **Age (years)** | **Pregnancy history** | **Gestational weeks at diagnosis (weeks)** | **Gender** | **Prenatal findings** | **Follow-up** | **Autopsy** | ***PKHD1* variants** | **Reference** |
| --- | --- | --- | --- | --- | --- | --- | --- | --- | --- |
| 1 | 33 | gravida l, para 0 | 36 | female | liver: two large cystic structures with scattered echogenic foci (central dot sign by ultrasound); kidney: enlarged and echogenic kidneys; others: Oligohydramnios | caesarean section at 36 weeks gestation; 1-min and 5-min Apgar scores of 1 and 1; Potter's facies; grossly distended right flank and abdomen; Newborn died shortly after birth | right kidney weighing 900 g and left kidney weighing 100 g; multiple cysts in both kindney (IPKD); cystic structures and vascular tracts within dilated biliary radicles in liver | - | Hussman, et al. 1991 (Hussman et al., 1991) |
| 2 | 28 | gravida 4 para 1 abortus 2 | 23 | female | liver: multiple cystic dilatations in the liver; kidney: a normal appearance; others: femur length was < 5th centile; mild polyhydramnios; normal karyotype | Vaginal delivery at 38 weeks of gestation; feeding problems and intermittent cyanosis around 22nd day of life; dilated intrahepatic bile ducts by ultrasound and CT: acholic stool passage; Uncontrollable convulsions and sepsis;  baby died around 11 weeks of hospitalization. | - | - | Yuksel et al., 2002 (Yuksel et al., 2002) |
| 3 | 33 | gravida l, para 0 | 33 | male | liver: multilocular lesion in the right lobe of the liver at 33 weeks’ gestation; kidney: right pyelectasis at 19 weeks’ gestation and resolution of the pyelectasis at 33 weeks’ gestation; | Vaginal delivery at term with no postnatal complications;  diffuse dilatation of bile ducts in both lobes and bilateral enlarged polycystic kidneys by ultrasound (ARPKD);  hyponatremia on day 3 of postnatal life, and then arterial hypertension; normal renal and liver functions during his first 3 months of life. | - | IVS55 + 1G → A;  W2690R | Sgro, et al., 2004 (Sgro et al., 2004). |
| 4 | 26 | gravida l, para 0 | 24 | not mentioned | liver: enlarged liver and saccular dilatations; no “central dot sign” by MRI; kidney: bilateral enlarged kidney with multiple cystic lesions (ARPKD); others: The bladder was not visible; oligohydramnios | The fetus died in utero at 26 weeks of gestation | - | - | Castro, et al., 2017 (Castro et al., 2017) |
| 5 | 28 | gravida 6, para 3 | 29 | male | liver: enlarged liver with multiple cystic structures; “central dot sign” by MRI; kidney: bilateral enlarged and echogenic kidneys, showing increased signal on by fetal MRI (ARPKD); others: oligohydramnios; | cesarean delivery at 34 weeks of gestation because of anhydramnios; labored breathing; left nephrectomy at 3 months of age; renal and liver transplantation at 16 months of age. The patient was doing well 1 year after the transplant. | - | c.8407T>C (p.C2803R) | Rivas, et al., 2019 (Rivas et al., 2019) |
| 6 | 37 | gravida l, para 0 | 28 | female | liver: dilated biliary tree; ‘central dot’ sign by MRI; Kidney: a normal appearance at 13 weeks of gestation; enlarged and hyperechogenic kidneys at 21 weeks (ARPKD); others: oligohydramnios; lung hypoplasia | Cesarean section at 35 + 2 weeks’ gestation due to breech presentation. The newborn expired 55 min after delivery because of severe respiratory insufficiency due to lung hypoplasia. | The autopsy confirmed the prenatal diagnosis and also showed liver fibrosis. | - | Castro, et al., 2020 (Castro et al., 2020) |
| 7 | 39 | gravida 2, para 0 | 33 | Female | liver: small tubular and cystic dilatations; ‘central dot’ sign by MRI; kidney: echogenic kidneys with loss of corticomedullary differentiation; multiple tiny cystic lesions by MRI (ARPKD); | The pregnancy was terminated. | - | c.7912T>A (p.Y2638N);  c.3364+3A>T | This study |
| 8 | 26 | gravida l, para 0 | 39 | Female | Liver: saccular anechoic spaces in the right lobe of the liver ; no visible ‘central dot’ sign by MRI; kidney: enlarged and echogenic kidneys; numerous cystic structures by MRI (ARPKD);  others: Oligohydramnios | Vaginal delivery at term; the baby died a few days after birth; the findings of the newborn by MRI confirmed the diagnosis of CD/CS with ARPKD. | - | c.9901G>T (p.E3301X); c.2507T>C (p.V836A) | This study |

-, not recorded or declined.

**References**

Castro, P., Werner, H., Matos, A.P.P., Peixoto-Filho, F.M., Andrade, C.V., and Araujo Júnior, E. (2020). Caroli's syndrome evaluated by ultrasound and magnetic resonance imaging during pregnancy. *Ultrasound Obstet Gynecol* 56(1)**,** 125-127. doi: 10.1002/uog.22016.

Castro, P.T., Matos, A.P.P., Werner, H., Daltro, P., Fazecas, T., Nogueira, R., et al. (2017). Prenatal Diagnosis of Caroli Disease Associated With Autosomal Recessive Polycystic Kidney Disease by 3-D Ultrasound and Magnetic Resonance Imaging. *J Obstet Gynaecol Can* 39(12)**,** 1176-1179. doi: 10.1016/j.jogc.2017.04.041.

Hussman, K.L., Friedwald, J.P., Gollub, M.J., and Melamed, J. (1991). Caroli's disease associated with infantile polycystic kidney disease. Prenatal sonographic appearance. *J Ultrasound Med* 10(4)**,** 235-237. doi: 10.7863/jum.1991.10.4.235.

Rivas, A., Epelman, M., Danzer, E., Adzick, N.S., and Victoria, T. (2019). Prenatal MR imaging features of Caroli syndrome in association with autosomal recessive polycystic kidney disease. *Radiol Case Rep* 14(2)**,** 265-268. doi: 10.1016/j.radcr.2018.11.006.

Sgro, M., Rossetti, S., Barozzino, T., Toi, A., Langer, J., Harris, P.C., et al. (2004). Caroli's disease: prenatal diagnosis, postnatal outcome and genetic analysis. *Ultrasound Obstet Gynecol* 23(1)**,** 73-76. doi: 10.1002/uog.943.

Yuksel, A., Has, R., Isikoglu, M., and Suoglu, O. (2002). Prenatal diagnosis of Caroli's disease. *Ultrasound Obstet Gynecol* 19(5)**,** 525-526. doi: 10.1046/j.1469-0705.2002.00692.x.
